# A short-term approach for promoting oral health of internally displaced children with PTSD: the key is improving mental health—results from a quasi-randomized trial

**DOI:** 10.1186/s12903-020-01385-z

**Published:** 2021-02-10

**Authors:** Sulaf Hamid, Mayssoon Dashash, Youssef Latifeh

**Affiliations:** 1 Department of Pediatric Dentistry, Faculty of Dentistry, Damascus University, Damascus, Syria; 2 Centre for Measurement and Evaluation in Higher Education, Ministry of Higher Education, Damascus, Syria; 3 Department of Psychiatry, Damascus University, Damascus, Syria; 4 Department of Psychiatry, Almouwasat University Hospital, Damascus University, Damascus, Syria

**Keywords:** Approach, Promoting oral health, PTSD, Children

## Abstract

**Background:**

Several studies have demonstrated that mental (MH) and oral health (OH) of displaced children are negatively affected during the wartime. This may be a result of general self-neglect and psychological suffering. Therefore, previous studies suggested that psychosocial support (PSS) is essential during and after humanitarian crises to prevent immediate and long-term MH and OH problems. This study was undertaken to evaluate the effectiveness of a short-term approach in improving (MH) and (OH) of displaced children suffering from posttraumatic stress disorder (PTSD).

**Methods:**

A quasi-randomized clinical trial study was carried out including (118) displaced children suffering from PTSD. The Child Post-Traumatic Stress Reaction Index (CPTSD-RI) was utilized for the diagnosis of PTSD. Children were assigned into two groups (intervention and control group). Children in the intervention group were enrolled in a 6-week PSS program that contained oral health educational components designed especially for this study. Clinical evaluation included plaque index (PI) and gingival index (GI). Oral health related Quality of life (OHRQoL) was also evaluated using child perception questionnaire (CPQ_11-14_). Study variables were evaluated at baseline and at the end of the program for both groups. Wilcoxon rank test and *t*-test for independent samples were used for data analysis.

**Results:**

A total of 118 children, aged between 9 and 14 years, participated in the recent study (mean age 11.0 ± 1.4). All participated children were previously diagnosed with PTSD. At baseline, there were no significant differences in the study variables between groups (*P* > 0.05). At the end of the program, children in the intervention group had significantly decreased PI, GI, CPQ_11-14_ and CPTSD-RI compared to their baseline scores (*P* = 0.000). In contrast, controls showed no differences at the end of the program (*P* > 0.05). Children in the intervention group had significantly (*P* = 0.000) lower PI (1.52 ± 0.55) and GI (1.48 ± 0.56) when compared to controls (PI = 1.89 ± 0.39, GI = 2.14 ± 0.32) post program. Moreover, the intervention group showed remarkable decline (*P* < 0.001) in their CPQ_11-14_ (47.16 ± 12.24) and CPTSD-RI (34.41 ± 12.23) scores compared to controls (CPQ_11-14 =_ 72.65 ± 14.47, CPTSD-RI = 47.91 ± 14.24) post program.

**Conclusions:**

The designed approach could have positive improvements in PTSD symptoms, (OH) and (OHRQoL) of displaced children. Integration between (MH) and (OH) services should be considered during and after humanitarian crises to prevent immediate and long-term MH and OH problems.

*Trial registration* Australian New Zealand Clinical Trials Registry (ACTRN12619000285156), Date registered: 25/02/2019, retrospectively registered. https://anzctr.org.au/Trial/Registration/TrialReview.aspx?id=377001&isReview=true.

## Background

Most of children exposed to ongoing stressors of war will experience significant psychological morbidity [1]. Moreover, displacement as a usual consequence of war, also force children and their families to live in unhealthy conditions of malnutrition, overcrowding and psychological uncertainty [2]. This is the case now for Syrian Families. While the war is about to terminate, still the post-war period seems to be even harder for Syrians. MH now is a major problem since outpatients have been dramatically increased in psychiatric clinic during the crisis [3]. Furthermore, OH is negatively affected. A recent study showed that gingivitis prevalence reached 97.93% among children during the crisis [4]. Unfortunately, OH has less attention by researchers and health policy makers during a wartime compared to MH. Still, OH can add fuel to fire for mentally ill people if they happen to encounter. A study found that oral symptoms may be the first or the only manifestation of (MH) problem that may affect QoL [5]. In another hand, previous studies have demonstrated that people with (MH) problems are prone to develop (OH) problems because of the general self-neglect resulting from their psychological suffering [6, 7]. Recently, WHO referred to MH as an integral part of public health programs and not a specialist activity setting apart [8]. In addition to the fact that integration between (MH) and non-specialized healthcare is a possible strategy to make targeted interventions sustainable [9]. The aim of the current study was to evaluate the effectiveness of short-term approach in improving (MH) and (OH) of displaced children suffering from posttraumatic stress disorder (PTSD).

## Methods

### Ethical considerations

This study was approved by the Ethical Committee of the Faculty of Dentistry in Damascus University, Syria. All children participated in the current study were informed, along with their parents, about the study aims and procedures. Parents/guardians have signed the written informed consent.

### Study design and sample

This study has a quasi-randomized controlled trial design. The study carried out in 2017, Damascus, Syria. During this year Damascus city had 8 temporary accommodation centers (TACs). These centers were set up by the government to receive displaced families coming from hot areas (around Damascus and different parts of Syria). Four TACs were selected from four different areas in Damascus city. All children aged between 9 and 14 years living in TACs were invited to participate.

### Study procedures

#### Randomization and sampling

**Fig. 1:**
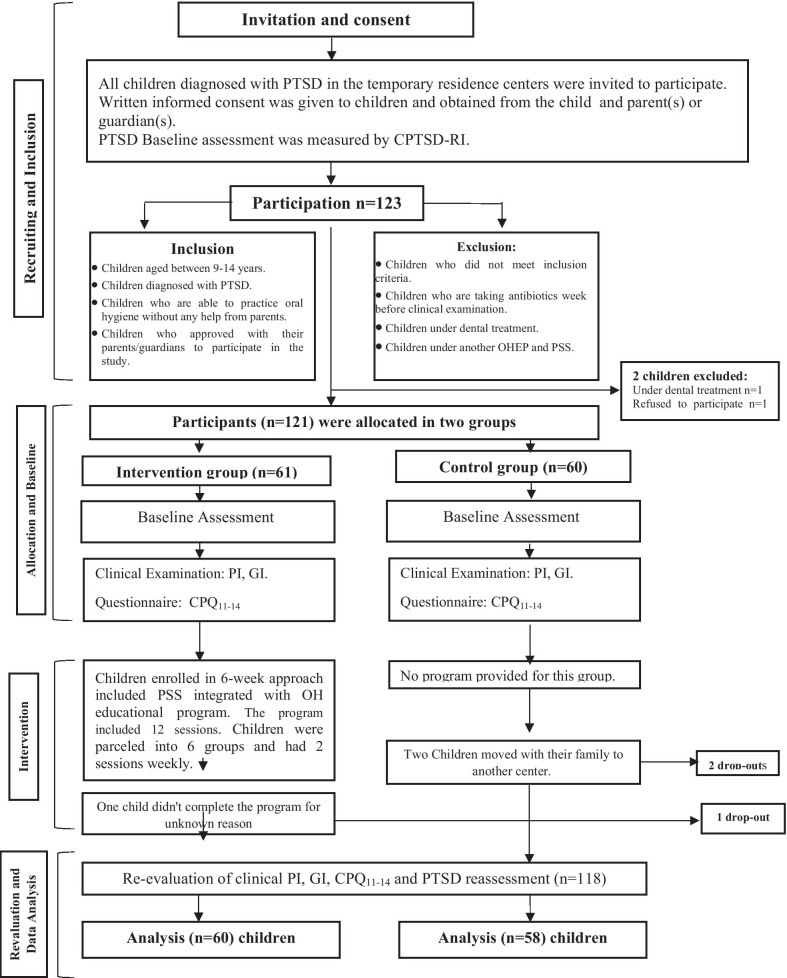
Flow chat of children participated in the study

Two TACs (out of four) were selected to apply the intervention (intervention group). The other two centers served as control group. Randomization for selecting TACs was utilized by means of coin toss. Inclusion and exclusion criteria are shown in Fig. 1. (The flow chart of children participated in this study). Clinical examination and questionnaires were evaluated at baseline and within one week at the end of the program (after the 6-week approach).

A total of 40 patients in each group were necessary to reach 80% of statistical power according to a previous similar study [10]. Therefore, a total sample size should include eighty subjects (N = 80). To allow adjustment of other factors such as withdrawals and missing data, additional subjects (20 subjects) were added to each group. Therefore, the study included an intervention group (n = 60) and a matching controls (n = 60).

#### Diagnosis of PTSD

Only children with PTSD participated in the recent study. PTSD was assessed prior to the intervention using CPTSD-RI. This self-report questionnaire had 20 items assessing PTSD symptoms among children aged between 6 and 16 years [11]. Previous studies showed that the Arabic version of this scale is reliable and valid [12]. High scores indicate worse symptoms.

#### OHRQoL assessment

OHRQoL was assessed using the CPQ_11-14_ self-report scale [13]. This scale has 36 questions about how oral symptoms, Functional limitation, emotional and social wellbeing are affected by OH. The Arabic version of the scale showed high reliability and validation [14].

Children were asked to complete the two questionnaires pre and post program. Each questionnaire took about 15–20 min to complete. Children were instructed to read and answer the questionnaire themselves. They were also informed about the possibility of withdrawing from the study at any point. A pediatric dentist (SH) and a psychologist (YL) was supervising this process and were available at any time to answer any question.

### Clinical examination

The same investigator (SH) held out the clinical examination of children in both groups. OH was evaluated using PI [15] and GI [16]. The indices were recorded for all children pre and post program. The clinical examination performed according to the basic methods and diagnostic criteria of WHO [17].

### Description of the approach

A short-term approach was designed specifically for this study. The 12-session program was carried out for 6 weeks. The program included 8 sessions of PSS and 4 sessions of educational materials about OH. The same pediatric dentist (SH) held the OHEP, while two well-trained social workers conducted PSS part of the program. The same psychologist (YL) supervised the whole sessions and was always available in case any children had serious symptoms and needed urgent referral. Children attended two sessions weekly in terms of sub-groups. The intervention group included 6 sub-groups. Each sub-group consisted of 18–20 children. For six weeks, the exact same session was conducted for two sub-groups in the first three days of the week. In the following three days, a new session was carried out. Every session took about 60–90 min. Sessions had various integrated activities such as playing, drawing, acting and relaxation methods. Educational materials about OH included presentations, videos, illustration tools and storytelling. PSS focused on trauma healing, open discussions about social problems, friendly behavior and positive vision about the future. The educational program focused on oral diseases and their consequences on General Health. It also focused on prevention methods, treatment procedures/tools, stages of teething and dental appearance. Children were also educated about the importance of OH to the whole body and how periodic checkups are necessary for their OH.

Children in the control group served as a comparison group to assess the spontaneous improvement -if any- of study variables without any intervention. It would be unethical to include only children in the intervention group participated in the designed approach. Therefore, children in the control group were also invited to experience the same exact program at the end of the study.

### Statistical analysis

Data were analyzed by means of SPSS program (SPSS 20, SPSS Inc. Chicago, IL, USA). Kruskall-Wallis test was used to check the normality distribution of the data. Data of the recent study weren't normally distributed. Therefore, Wilcoxon signed rank test was used to assess the mean changes over time pre/post program in the same group. Independent sample *t*-test was used to measure differences between the intervention and the control group pre/post program. The *P* value for all tests was set at 0.05.

## Results

A total of 118 children aged 9–14 years (mean age was 11.0 ± 1.4 years) participated in this study. The intervention group (n = 60) included 38.3% boys and 61.7% girls. The controls (n = 58) included 34.5% boys and 65.5% girls. Table 1 presents the distribution of participated children according to demographic characteristics and group.

**Table 1:** Demographics of PTSD children in both intervention and control groups

Groups		Intervention		Control		Total	
Variables		n	%	n	%	N	%
Gender	Boy	23	38.3	20	34.5	43	36.4
	Girl	37	61.7	38	65.5	75	63.6
Total		60	100	58	100	118	100
Age	mean ± SD	10.9 ± 1.4		11.1 ± 1.4		11.0 ± 1.4	

SD, Standard Deviation

Table 2 presents the PI, GI, CPQ_11-14_ and CPTSD-RI for children in intervention and control group pre and post program. Findings revealed no significant differences (*P* > 0.05) between the intervention and the control groups regarding baseline scores for all study variables. Post program children in the intervention group showed significantly (*P* = 0.000) lower scores regarding PI (1.52 ± 0.55) and GI (1.48 ± 0.56) compared to the controls (PI = 1.89 ± 0.39, GI = 2.14 ± 0.32). Their CPQ_11-14_ score was also significantly lower (47.16 ± 12.24) compared to the controls (72.65 ± 14.47). Moreover, the CPTSD-RI have been significantly (*P* = 0.000) decreased post program in the intervention group (34.41 ± 12.23) compared to controls (47.91 ± 14.24).

**Table 2:** Differences between PTSD children in intervention and control group according to oral (PI, GI) and mental (CPQ_11-14,_ CPTSD-RI) health variables pre and post program

Variable		Intervention			Control			Differences**
		mean	SD	*P* value*	mean	SD	*P* value*	
PI	Pre	2.15	0.64	0.000	1.97	0.53	0.035	0.114
	Post	1.52	0.55		1.89	0.39		0.000**
GI	Pre	2.13	0.45	0.000	2.14	0.50	0.873	0.927
	Post	1.48	0.56		2.14	0.32		0.000**
CPQ_11-14_	Pre	74.80	15.97	0.000	75.29	12.30	0.175	0.852
	Post	47.16	12.24		72.65	14.47		0.000**
CPTSD-RI	Pre	45.66	14.86	0.000	45.81	13.94	0.075	0.957
	Post	34.41	12.23		47.91	14.24		0.000**

PI, Plaque Index; GI, Gingival Index; CPQ_11-14_, Child Perception Questionnaire age between 11 and14; CPTSD-RI, Child Posttraumatic Stress Reaction Index; SD, Standard Deviation

*Wilcoxon Signed Ranks Test

**Independent samples t-test

Table 3 presents the differences between boys and girls in intervention group according to all health variables pre and post program. Baseline scores showed that boys and girls had similar scores with no significant differences regarding study variables (*P* > 0.05). Post program, both boys and girls in the intervention group revealed significant (*P* < 0.05) decline in all study variables. However, girls had significantly lower PI (1.34 ± 0.57) compared to boys (1.81 ± 0.39) post program. Moreover, girls also showed significantly (*P* = 0.023) lower CPTSD-RI score (31.62 ± 12.23) compared to boys (38.91 ± 11.04) post program.

**Table 3:** Differences between boys and girls in intervention group according to oral (PI, GI) and mental (CPQ_11-14,_ CPTSD-RI) health variables pre and post program

Variable		Boys			Girls			Differences**
		mean	SD	*P* value*	mean	SD	*P* value*	
PI	Pre	2.28	0.45	0.000	2.06	0.73	0.000	0.217
	Post	1.81	0.39		1.34	0.57		0.000**
GI	Pre	2.05	0.43	0.000	2.18	0.45	0.000	0.296
	Post	1.53	0.55		1.45	0.56		0.602
CPQ_11-14_	Pre	73.82	16.67	0.000	75.40	15.71	0.000	0.716
	Post	48.69	12.38		46.21	12.22		0.452
CPTSD-RI	Pre	45.56	13.12	0.001	45.72	16.03	0.000	0.967
	Post	38.91	11.04		31.62	12.23		0.023**

PI, Plaque Index; GI, Gingival Index; CPQ_11-14_, Child Perception Questionnaire age between 11 and 14; CPTSD-RI, Child Posttraumatic Stress Reaction Index; SD, Standard Deviation

*Wilcoxon Signed Ranks Test

**Independent samples t-test

## Discussion

The aim of psychosocial interventions inside wider developmental contexts such as healthcare is to create an integrated developmental approach to promoting psychosocial wellbeing. Integrative approaches of education on health and psychosocial support must be accompanied by more efforts. In this way, the psychosocial wellbeing of children can be assured [18]. Therefore, the aim of the current study was to evaluate the effectiveness of a novel approach in improving both MH and OH of children traumatized by war. For this purpose, children suffered from PTSD were enrolled into a short-term approach designed especially for this study. The Designed approach provided PSS integrated with OHEP.

The recent study had a quasi-randomized trial for financial and practical constrains related to the war circumstances. Children were randomly selected from four temporary accommodations centers. Children related to two centers, were allocated to intervention group whilst children related to other two centers were allocated to control group. By separating the intervention and the control groups, the contamination between study participants was controlled.

The used approach had positive effects on children suffering from PTSD after participating in the study program. Children enrolled in the program showed significant improvement in their PTSD symptoms. They had better OH status and OHRQoL post program compared to baseline scores. On the other hand, children in the control group showed no or slight improvement and sometimes worse scores post-program. This can be explained by the fact that those children were still under the stressor and distress of war without any kind of PSS.

In this study, the short-term approach was provided to traumatized Syrian children affected by the war. Most previous studies on war children focused more on treatment techniques utilized by psychologists rather than PSS provided by non-specialists [18]. Unfortunately, MH service in Syria have not yet been integrated into public health care strategies. Therefore, governmental and nongovernmental associations have trained social worker personnel to provide PSS to affected and displaced children. Nonetheless, no previous studies have reported the effectiveness of such services.

A significant reduction in PTSD symptoms among intervention group was reported. Dybdahl found Similar findings in 2001. He concluded that group-PSS had significantly improved MH of internally displaced children post war. The program integrated with basic medical care. The study also involved mothers of the children [19]. In the contrary, another study did not find significant impact of group intervention provided to Palestinian children suffering from PTSD. The authors attributed this to the continuing exposure to trauma and non-active nature of the intervention [10].

It is worth mentioning that the recent study focused more on PSS part of the approach rather than on the OHE. Integrated OHE was a way to change the traditional method of providing PSS into enjoyable and useful process rather than focusing on trauma itself. The plaque and gingival indices were used to evaluate changes in the OH status. Future longitudinal studies that consider other OH evaluations could be used to investigate changes over time. In the recent study, both PI and GI have significantly declined among children in the intervention group post program. A recent study (2018) conducted on 220 schoolchildren aged between 10 and 11 years in Syria showed that Plaque and Gingival Indices were improved after 6 weeks of educational program on OH compared to baseline [20]. However, the study did not study the psychological well-being of the children and did not provide a PSS.

Girls in the recent study showed lower PI compared to boys pre and post program. They also showed significant improvement in PTSD symptoms compared to boys post program. A previous study found similar results after school-based MH intervention carried out in conflict-affected areas in Indonesia [21].

## Study limitations

Previous studies have focused only on general health when providing PSS integrated with healthcare. This novel approach was the first of its kind in the medical literature trying to add outcomes regarding OH promotion through PSS during humanitarian emergences. Still, this study has some limitations. One of the limitations was that PTSD diagnosis relied only on the self-report questionnaire and not on structured interview with a psychologist. Another limitation was that the same investigator conducted the clinical examination post program, which may influenced the objectivity of the assessment. Moreover, the TACs in Damascus city were all closed after a short time of our study. Most of the participated children had left the TACs to their original residence as a part of the "coming back home" plan established by the government post war. Therefore, tracing the long-term effects of our short-term approach was not available.

## Conclusions

The improvement of both oral and mental health of displaced children was observed after a short-term approach focused in PSS and OHE. The designed approach had positive improvements in PTSD symptoms, (OH)) and (MH) of displaced children. This proves that integration between MH and OH services can be suggested during and after humanitarian crises to prevent immediate or long-term MH and OH problems. We believe that, dentists, as health care professionals, have an obligation to play a role in health care strategies and interventions such as PSS. Therefore, these kinds of interventions may improve overall health outcomes and lead to a healthier society.

## Data Availability

The datasets used and/or analyzed during the current study are available from the corresponding author on reasonable request.

## Abbreviations

PTSD: Posttraumatic stress disorder
CPTSD-RI: Child posttraumatic stress reaction index
PSS: Psychosocial support
OHEP: Oral health educational program
OH: Oral health
MH: Mental health
OHRQoL: Oral health related Quality of life
PI: Plaque index
GI: Gingival index
CPQ_11-14_: Child perception questionnaire from 11 to 14 years
TACs: Temporary accommodation centers
SD: Standard Deviation
SH: Sulaf Hamid
MD: Mayssoon Dashash
YL.: Youssef Latifeh
